# Validity and Reliability of Resting Energy Expenditure Measured by Indirect Calorimetry in Adults with Overweight and Obesity: a Rapid Systematic Review

**DOI:** 10.1007/s11695-025-08220-w

**Published:** 2025-09-09

**Authors:** William Bruce, Lynette Law, Elizabeth Chen, Xueying Tang, Skye Marshall

**Affiliations:** 1 Faculty of Health Sciences & Medicine, Bond University, Gold Coast, Australia; 2 La Trobe Sport and Exercise Medicine Research Centre, School of Allied Health, Human Services and Sport, La Trobe University, Melbourne, Australia; 3 Centre for Quality and Patient Safety Research, Institute for Health Transformation, School of Nursing and Midwifery, Faculty of Health, Deakin University, Burwood, Australia

**Keywords:** Overweight, Obesity, Indirect calorimetry, Energy metabolism, Systematic review, Psychometrics

## Abstract

This rapid systematic review aimed to evaluate the diagnostic accuracy (concurrent validity, predictive ability, reliability) of indirect calorimetry (IC) for measuring resting energy expenditure (REE) in adults with overweight or obesity. PubMed and Web of Science searched for studies measuring REE by IC in adults with overweight or obesity and reported primary outcomes: concurrent validity, predictive ability, or reliability. *N* = 22 studies were included that evaluated *n* = 10 IC devices. A handheld IC device was reported to have poor concurrent validity and poor reliability. Standard desktop IC devices were reported to have inconsistent concurrent validity, inconsistent predictive ability for weight loss, and good to excellent reliability. Whole-room IC devices were reported to have excellent reliability. Further research is needed.

**Supplementary Information:**

The online version contains supplementary material available at 10.1007/s11695-025-08220-w.

## Introduction

The global prevalence of overweight (BMI 25–29.9 kg/m^2^) and obesity (BMI ≥ 30 kg/m^2^) has increased significantly in the past three decades with more than a two-fold increase in prevalence in adults [1]. The overreliance on energy-dense, nutrient-poor foods and beverages, reduced physical activity, urbanization, and sedentary recreation options, has been found to contribute to the increased rates of overweight and obesity [2]. Elevated BMI is a modifiable risk factor for chronic medical conditions such as cardiovascular disease, stroke, and diabetes, rates of which have increased and have been identified as leading contributors of mortality globally [3]. The consequences of overweight and obesity go beyond typical definitions of health, contributing to reduced economic productivity and increased reliance on social security, evidenced by increased rates of absenteeism [4]. Existing treatment approaches to manage overweight and obesity encompass dietary behavior change, regular physical activity, medications, and metabolic-bariatric surgery (MBS).

With knowledge of this growing healthcare crisis, research has shown that accurate assessment of an individual’s energy requirements may increase the efficacy of weight loss interventions [5–7]. Energy requirement refers to the daily caloric intake required to maintain body weight and is directly related to the Total Energy Expenditure (TEE), which fluctuates highly based on physical activity, illness, diet-induced (DIT) thermogenesis, a prolonged energy deficit or surplus [8], and resting energy expenditure (REE) [9]. REE is the largest contributor to TEE and is affected by age, sex, body size, body composition, and genetics [9]. Without accurate measurement of resting energy expenditure (REE), there is risk of over- or under-estimating the energy requirements of individuals, thus undermining weight and comorbidity management strategies for patients with overweight and obesity [10]. In clinical practice, REE may be estimated using predictive equations; however, these have been found to be unreliable in individuals with extremes of BMI [11]. Indirect calorimetry (IC), which measures the exchange of carbon dioxide and oxygen to assess REE, has been considered as the best practice non-invasive option for determining REE in patients with overweight or obesity [11].

Despite the potential for IC to provide an accurate measure of REE for adults with overweight or obesity, evidence on the validity and reliability of IC devices has not been previously synthesized. This study aimed to examine the validity, predictive ability, and reliability of IC compared to all other forms of REE measurement in adults with overweight and obesity.

## Materials and Methods

A rapid systematic review was conducted by adopting elements of Cochrane Rapid Review Methods Recommendations: (1) reduced number of electronic databases searched, (2) exclusion of non-English papers, (3) targeted research question, and (4) exclusion of grey literature [12]. To maintain the rigor of the data presented, record selection and assessment of risk of bias were conducted in duplicate.

This review was reported using the Preferred Reporting Items for Systematic Reviews and Meta-Analysis (PRISMA) 2020 [13] Statement and prospectively registered with the International Prospective Register of Systematic Reviews (PROSPERO number: CRD42024505339).

### Search Strategy

An electronic search of MEDLINE via PubMed and Web of Science for peer-reviewed studies in the English language was performed from inception to June 2025. The search strategy combined keywords (title and abstract) and Medical Subject Headings (MeSH) terms related to indirect calorimetry, validity, and reliability (Table S1). A snowball search was conducted to complement the systematic search using the reference lists of the included studies and papers on similar topics.

### Eligibility Criteria

Eligible studies have been outlined according to the PICOS framework (Table 1). For the review, “desktop” IC devices were defined as those designed primarily for stationary lab or clinical use with larger form factors, often including a cart-based system (e.g., metabolic carts), and “portable” devices were defined as self-contained units intended for field or point-of-care use. For studies which did not exclusively include a sample with overweight or obesity (BMI ≥ 25kg/m^2^), a mean BMI of ≥ 30 kg/m^2^ was selected to ensure most of the sample would represent adults with overweight and obesity. In studies where results were presented according to BMI category, only the subsamples of overweight (BMI 25.0–29.9kg/m^2^) or obesity (BMI ≥ 30kg/m^2^) were eligible.

**Table 1:** Eligible studies according to the Participant, Indicator, Comparator, Outcome, and Study design (PICOS) framework

	Included	Excluded
Participant	Sample of adults with a mean age of 18 + years and either (a) whole sample/sub-sample BMI ≥ 25 kg/m^2^ or (b) mean BMI ≥ 30 kg/m^2^	Animal studies
Indicator	IC device that measured REE^a^ via gas exchange while the participant is at rest [14, 15]	IC device that measured REE^a^ using body heat or obtained while the participant is active or sleeping
Comparator	For validity: measurement of energy requirements, metabolic rate, or energy expenditure whilst at rest	For validity: predicted energy requirements, metabolic rate, or energy expenditure, total energy expenditure whilst not at rest or over an extended period of time, e.g., doubly labelled water
Outcome	One or more primary outcomes Validity: any accuracy tests (e.g., criterion, comparative, etc.) Reliability: re-tests (agreement) Predictive: any health outcome measured prospectively according to RRE/BMR	
Study design	Validity or reliability outcomes: cross-sectional or cohort studies. Prospective ability: prospective or retrospective cohort studies	Non-peer reviewed research, protocols, systematic reviews, full text unavailable, and conference abstracts. Non-English publications

*BMI* body mass index, *IC* indirect calorimetry, *REE* resting energy expenditure

^a^Concept alternative nomenclature for REE were eligible, including basal metabolic rate (BMR), basal energy expenditure (BEE), and resting metabolic rate (RMR); however, for consistency, were referred to throughout this review as REE

### Selection of Studies

Identified records from both databases were imported into EndNote [Version 21.2, Clarivate], and duplicates were removed. A two-step selection process was employed utilizing Covidence software [16]. In step 1, two investigators independently evaluated the titles and abstracts of records identified by the search for their potential eligibility. At step 2, potentially eligible articles were assessed via full text by two investigators independently. Disagreements regarding eligibility were discussed to reach consensus or were decided by a third investigator.

### Outcomes and Data Management

Criterion validity is composed of two types of validity assessment: concurrent and predictive. Concurrent validity is determined by comparing the score of a new measurement to the score of a well-established measurement, or gold standard, for the same construct. Data extracted to reflect the concurrent validity were measures of diagnostic accuracy tests, including sensitivity, specificity, or agreement, for REE, with or without respiratory quotient (RQ; measure calculated by VO_2_/VCO_2_) [17]. The reference standard used for comparison was described. Where possible, we designated devices with greater methodological rigor or regulatory recognition as the reference standard. In cases where the study authors did not designate a reference device, we made this judgement based on technical characteristics and common usage patterns. Predictive validity may be evaluated by determining the ability of an assessment to predict health-related outcomes known to be related to the condition measured. In this circumstance, the REE in adults with overweight or obesity can be evaluated for weight-related outcomes such as weight or body composition change [18]. Weight-related outcomes were considered only if they were measured subsequently to the assessment of the REE via IC, with a follow-up timeframe from 1 week to 10 years. Reliability of a device was evaluated via agreement between assessment operators (inter-rater reliability) or agreement with multiple measures (intra-rater reliability). All data were described narratively in tables. Data were extracted by a single investigator and checked for accuracy and completeness by another investigator. Due to high clinical heterogeneity, data were not meta-analyzed.

### Review of Study Strength and Quality

Internal study quality (including risk of bias) for included studies was evaluated by the Critical Appraisal Skills Programme (CASP) Diagnostic Checklist [19]. The tool contained 12 questions with nine being able to be answered with “yes,” “no,” or “can’t tell.” The appraisal of study quality was conducted independently by a single investigator and checked for agreement by another investigator. Disagreements were discussed until consensus was reached. Risk of bias was noted in the results tables as either “low,” “moderate,” or “high.”

## Results

### Search Results and Included Studies

The search identified *n* = 4956 unique (deduplicated) records (Figure S1). After title and abstract screening, *n* = 580 were assessed via full text for eligibility and *n* = 22 were included: *n* = 10 evaluated concurrent validity [20–29], *n* = 7 evaluated predictive validity/ability [7, 30–35], and *n* = 8 evaluated reliability [20, 22, 27, 36–40] (Table 2). Three studies contributed data to more than one outcome type [20, 22, 23]. Studies were included from North America (*n* = 11 studies), Europe (*n* = 6 studies), South America (*n* = 4 studies), and Asia (*n* = 1 study), and had samples that ranged from *n* = 4 to *n* = 233, with most studies having a majority (> 50%) of females (*n* = 17; 77%). Most studies included community samples (*n* = 6) or patients seeking non-surgical weight loss (*n* = 6), followed by MBS cohorts (*n* = 5) or a mix of MBS and other participant types (*n* = 3), chronic disease (*n* = 1), or post-surgery intensive care unit (ICU; *n* = 1). All *n* = 22 studies followed similar assessment conditions with participants rested and fasted overnight, with most measurements being taken in the supine position (Tables 3–5). Detailed descriptions of devices used to measure REE can be found in the supplementary material (Table S3-5).

**Table 2:** Synthesis of the indirect calorimetry devices examined for validity and reliability in the measurement of resting energy expenditure in adults with overweight or obesity

Descriptor	Number (%)
Devices evaluated for concurrent validity (*n* = 10 studies)	
Device name measured for validity:	
- MedGem (handheld portable IC)	4 (40)
- Deltatrac (standard desktop IC)	2 (20)
- Fitmate (standard desktop IC)	2 (20)
- KORR-MetaCheck (standard desktop IC)	1 (10)
- COSMED Quark (standard desktop IC)	1 (10)
Device used as a comparator:	
- Vmax 29 N (standard desktop IC)	2 (20)
- OMRON HBF 500 (BC monitor)	1 (10)
- SenseWear (armband accelerometer)	1 (10)
- Deltatrac (standard desktop IC)	3 (30)
- InBody 720 (Bioimpedance)	1 (10)
- Whole-room IC	1 (10)
- Theoretical physical range	1 (10)
Devices evaluated for predictive ability (*n* = 7 studies)	
Device measured for predictive ability:	
- Vmax 29 N (standard desktop IC)	2 (29)
- Deltatrac (standard desktop IC)	3 (43)
- SensorMedics (standard desktop IC)	1 (14)
- MedGraphics (standard desktop IC)	1 (14)
Prospective health outcome evaluated:	
- Weight loss	6 (86)
- Weight regain	1 (14)
Devices evaluated for reliability (*n* = 8 studies; *n* = 10 comparisons)	
Device evaluated for reliability:	
- Vmax 29 N (standard desktop IC)	2 (20)
- Whole-room IC	1 (10)
- Deltatrac (standard desktop IC)	1 (10)
- MedGem (handheld portable IC)	2 (20)
- MedGraphics (standard desktop IC)	1 (10)
- Fitmate (standard desktop IC)^a^	1 (10)
- MM2B (standard desktop IC)	1 (10)
- Cosmed Q-NRQ + (standard desktop IC)	1 (10)

*BC* body composition, *IC* indirect calorimetry

^a^Fitmate device was considered standard desktop IC as it can be used with a ventilated hood/canopy system and not a mask

**Table 3:** Concurrent validity of indirect calorimetry to determine basal metabolic rate or resting energy expenditure evaluated in adults with overweight or obesity (*n* = 10 studies)

Study	Sample	Assessment context and conditions	Indirect calorimetry	Comparator	Outcome^a^	Reviewers’ conclusion	CASP risk of bias
Concurrent validity of a portable handheld indirect calorimeter							
Anderson et al. (2014) [20] USA	Total *N* = 88 (µ52 [SD: 8] y; 74% F; µ31.8 [SD: 4.2] kg/m^2^) Subgroup: OW NR Subgroup: OB NR	Community sample Rested, fasted state, reclined position	Device: MedGem, handheld IC	Device: Vmax 29 N (SensorMedics) IC (canopy hood, metabolic cart)	REE compared via paired t-test: 111.1 kcal/d difference, *p* < 0.0001	MedGem 6% higher REE than Vmax 29 N tending to over-estimate—some validity as overestimation may not be clinically relevant	Low
Cooper et al. (2009) [21] USA	Total *N* = 16 (µ49 [SD: 9] y; 100% F; µ47.9 [SD: 7.2] kg/m^2^) Subgroup: OW *n* = 0 Subgroup: OB N/A	MBS preoperative assessment Assessment after fasting overnight stay at research facility	Device: MedGem (Microlife USA, Golden, CO), handheld IC	Device: Deltatrac II Metabolic Monitor (VIASYS Healthcare, Inc., SensorMedics, Yorba Linda, CA)	REE compared via paired *t*-test: WSD µ − 121 (SD: 199) kcal/D, *p* > 0.05; CV µ7.7%, *p* > 0.05 REE compared via Bland–Altman: *R*^2^ = 0.31, *p* = 0.02	MedGem lacks validity compared to Deltatrac IC	Low
Frankenfield and Coleman (2013) [22] USA	Total *N* = 100 (µ44 [SD: 15] y; 84% F; µ33.4 [SD: 9.7] kg/m^2^) Subgroup: OW *n* = 0 Subgroup: OB *n* = 60 (µ50 [SD: 12] y; %F NR; µ40.2 [SD: 6.2] kg/m^2^)	Community sample Fasted, rested state, supine or semi-recumbent position	Device: MedGem (Microlife, Golden, CO), handheld IC	Device: Deltatrac Monitor (Deltatrac MB101; Deltatrac, Yorba Linda, CA), open circuit IC	REE compared via ANOVA: 119 (95%CI 67–171) kcal/day difference in supine Deltatrac and 171 (95%CI 116–227) kcal/d difference in semi-recumbent Deltatrac, *p* = NR REE compared via Bland–Altman: proportional bias for MedGem to overestimate REE (data not shown), *p* = NR	MedGem overestimated REE compared to Deltatrac, overestimation may not be clinically relevant, and statistical significance NR	Low
Purcell et al. (2020) [23] Canada	Total *N* = 26 (µ34 [SD: 9] y; 77% F; median 39.2 [IQR: 36.8–47.6] kg/m^2^) Subgroup: OW NR Subgroup: OB NR	MBS preoperative assessment Rested, fasted state, semi-supine position	Device: MedGem (Microlife Medical Home Solutions, Inc, San Jose, CA, USA), portable handheld IC	Device: Vmax 29 N (Senor-Medics, Yorba Linda, CA, USA), metabolic cart, ventilated hood	REE compared via paired *t*-tests: − 84 (95%CI − 504, 336) kcal/d difference, *p* = 0.057 REE compared via correlation: *r* = − 0.016, *p* = 0.940	MedGem REE was not different than metabolic cart REE on a group level	Low
Concurrent validity of a standard indirect calorimeter							
De Oliveira et al. (2012) [24] Brazil	Total *N* = 48 (µ26 [SD: 5] y; 0% F; µ29.3 [SD: 2.6] kg/m^2^) Subgroup: OW NR Subgroup: OB NR	Community sample Fasted, regular sleep–wake cycle 3 days before assessment, rested state	Device: KORR-MetaCheck device (Metabolic Rate Analysis System, model 7100, KORR Medical Technologies), portable IC	Device: Deltatrac-R3D (Deltatrac II, MBM-200, Datex Instrumentarium Corporation), metabolic cart	REE compared via ICC: ICC 0.561 (95%CI: 0.162–0.770); underestimated by 10.5% REE compared via Bland–Altman: µ201.3 (SD: 1.96)	KORR-MetaCheck had unreliable validity against the Deltatrac-R3D	Moderate
Bentes et al. (2021) [25] Brazil	Total *N* = 40 (µ63 [SD: 8] y; 100% F; µ30.9 [SD: 5.8] kg/m^2^) Subgroup: OW NR Subgroup: OB NR	Postmenopausal, metabolic syndrome Rested, fasted state	Device: Fitmate Pro (COSMED, Rome, Italy), portable IC	Device: InBody 720 (Biospace, Seoul, Korea), octopolar bioimpedance (BIA)	REE compared via intra-class coefficient of correlation: ICC = 0.906 (0.822–0.950), *p* = 0.001 After 6 months, ICC = 0.909 (0.829–0.952), *p* = 0.001 REE compared via Bland–Altman: Limits of agreement − 11.6 to 11.9, mean difference 0.1. After 6 months, limits of agreement –11.4 to 10.6, mean difference 0.4	High agreement in REE between IC and BIA estimates	Low
Popp et al. (2020) [26] USA	Total *N* = 63 (µ 54 [SD: 13] y; 75% F; µ35 [SD: 5] kg/m^2^) Subgroup: OW NR Subgroup: OB NR	Baseline assessment for weight loss trial participants (MBS or dietary restriction) Rested, fasted state, lay supine for 10 min	Device: COSMED Quark RMR metabolic cart (COSMED, Rome, Italy), IC with canopy hood	Range of RQ compared to the physiological range	RQ range: 0.65–1.2 was slightly outside physiological range (0.67–1.3), *p* = NR	Cannot conclude as valid as no true reference and slightly outside of physiological range	Low
Purcell et al. (2020) [27] Canada	Total *N* = 77 (median 30 [IQR: 26–37] y; 64% F; median 31.2 [IQR: 23.3–39.2] kg/m^2^) Subgroup: OW NR Subgroup: OB NR	Community sample and MBS sample (unclear if preoperative or postoperative) Rested, fasted state, supine position	Device: Fitmate GS (COSMED, Chicago, IL, USA), canopy hood, portable IC	Device: Whole body indirect calorimeter	REE compared via Bland–Altman: − 240 (95%CI − 727, 246) kcal/d, *p* = 0.140 REE compared via correlation: *r* = − 0.169, *p* = 0.140	Fitmate underestimated REE compared to whole body calorimetry and had poor accuracy compared to whole body indirect calorimetry	Low
Baudrand et al. (2013) [28] Chile	Total *N* = 60 (µ37 [SD: 13] y; 80% F; µ33.9 [SD: 6.1] kg/m^2^) Subgroup: OW NR Subgroup: OB NR	Outpatient sample from an overweight and obesity clinic Fasted sample	Device: Deltatrac MBM-100 (Datex Instrumentarium Corp, Helsinki, Finland), IC	Device: OMRON HBF 500 (Omron Corp, Kyoto, Japan), body composition monitor	REE compared via correlation: *r* = 0.87, *p* < 0.001	High correlation in REE between IC and body composition monitor estimates of REE	Low
Elbelt et al. (2010) [29] Germany	Total *N* = 78 (µ46 [SD: 12] y; 71% F; µ BMI: NR) Subgroup: OW NR Subgroup: OB *N* = 50 (µ 42–48 [SD: 11–13] y; 78% F; µ32.5–48.2 [SD: 1.3–5.3] kg/m^2^)	MBS preoperative assessment or non-alcoholic steatohepatitis outpatients Fasted, rested state, supine position	Device: Deltatrac II (Datex-Ohmeda, Freiburg, Germany), ventilated hood IC	Device: SenseWear armband accelerometer (InnerView Professional, Version 6.1, SMT medical technology, Wuerzburg, Germany), accelerometer	Difference in REE correlated with mean REE of both methods show higher disagreement in higher-BMI groups (*r* = − 0.268, *p* = 0.034)	SenseWear accelerometer had very weak validity compared to Deltatrac II in obesity subgroups	Low

*CASP* Critical Appraisal Skills Programme, *CV* coefficient of variation, *F* female, *kcal/day* kilocalories per day, *MBS* metabolic-bariatric surgery, *NR* not reported, *OB* obesity, *OW* overweight, *PCOS* polycystic ovary syndrome, *REE* resting energy expenditure, *RER* respiratory exchange ratio, *RMR* resting metabolic rate, *RQ* respiratory quotient, *SD* standard deviation, *USA* United States of America, *VO*_*2*_ volume of oxygen consumption, *VCO*_*2*_ volume of carbon dioxide produced, *WSD* within subject difference, *y* year, *µ* mean

^a^Intra-class correlation coefficient (ICC) often used for comparison testing. ICC of 1 represents perfect agreement, and a value of 0 means that there is no agreement at all. ICCs of 0 to 0.2 typically indicate poor agreement; 0.3 to 0.4 indicate fair agreement; 0.5 to 0.6 indicates moderate agreement; 0.7 to 0.8 indicates strong agreement; and > 0.8 indicates almost perfect agreement

### The Concurrent Validity of Indirect Calorimetry in Adults with Obesity

There were *n* = 6 IC devices evaluated for concurrent validity across *n* = 10 studies (Table 2) [20, 21, 23–29, 41]. Six studies compared a novel IC against a well-established standard desktop IC (*n* = 5) or a whole-room IC (*n* = 1). The remaining *n* = 4 studies used alternative measures of REE as comparators, including a bioimpedance device (*n* = 1), the respiratory quotient physiological range (*n* = 1), an accelerometer (*n* = 1), and a body composition monitor (*n* = 1) (Table 2). Risk of bias was assessed as low to moderate (Table 3).

The handheld MedGem IC was evaluated in *n* = 4 studies, all compared to standard desktop ICs (Deltatrac *n* = 2; Vmax 29 N *n* = 2) (Table 3). MedGem was found to overestimate REE by three studies with differences in measurements ranging from 111 to 171 kcal/day [20–22]. Whilst the fourth study evaluating MedGem reported no difference in kcal between the devices, the measure was imprecise (95%CI − 504 to 336 kcal/day) suggesting the sample of 26 participants was underpowered, decreasing confidence in the finding [27]. Overall, portable indirect calorimeters had poor concurrent validity compared to desktop IC devices due to clinically and/or statistically significant differences in measured REE (Table 3).

None of the *n* = 6 standard-desktop devices evaluated used a similar comparator (Table 3). The KORR-MetaCheck IC (*n* = 1 study) had unreliable validity compared to the Deltatrac standard-desktop IC [24]. The Fitmate GS IC underestimated REE by − 201.3 kcal/day compared to a whole-room IC [23], but the Fitmate Pro IC had high agreement with an octopolar bioimpedance device (InBody 720) [25]. The COSMED Quark was only compared against the theoretical physiological range for respiratory quotient, against which it was slightly out of range [26]. There was strong correlation in REE measured by the Deltatrac MBM-100 IC with a body composition monitor (*r* = 0.87; *p* < 0.001) [26]. However, the Deltatrac II IC displayed increasingly greater disagreement with higher BMI subcategories compared to the SenseWear armband accelerometer [29]. Notably, not all of the 78 participants in this study received dual measurements from both devices, reducing the sample size available for comparison [29] (Table 3).

### Predictive Ability of Indirect Calorimetry in Adults with Obesity

The predictive ability of IC for weight loss was evaluated by six studies [7, 30–33], and a seventh study by Wu et al. [39] examined predictive ability for weight regain, all of which were standard desktop IC devices (Table 2). The risk of bias ranged from low to moderate (Table 4). When examining the ability to predict weight loss from 10-week to 5-year follow-up, *n* = 4 reported no or weak associations [30–33]. One of the *n* = 3 studies that evaluated the Deltatrac reported that it was able to accurately predict weight loss at 1-year follow-up, where higher REE was associated with greater weight loss [34]. Another study reported that an unnamed IC (which may be the Vmax) demonstrated that dietary energy intake prescriptions based on measured REE resulted in greater weight loss as compared to energy intake based on estimated equations [7]. The only study to evaluate the MedGraphics for predictive ability [35] reported that changes in REE were not associated with future weight regain at 6- or 12-month follow-up.

**Table 4:** Predictive ability of indirect calorimetry to determine basal metabolic rate or resting energy expenditure evaluated in adults with overweight or obesity (*n* = 7 studies)

Study	Sample	Assessment conditions	Indirect calorimetry	Health outcome	Predictive ability	Reviewers’ conclusion	CASP risk of bias
Das et al. (2003) [30] USA	Total *N* = 30 (µ39 [SD: 10] y; 80% F; µ48.3 (F) [SD: 8.2] kg/m^2^)–57.3 (M) [SD: 8.2–10.4] kg/m^2^) Subgroup: OW NR Subgroup: OB NR	MBS preoperative assessment Resting, fasted state, supine position	Device: Deltatrac (SensorMedics Corp, Yorba Linda, CA), portable metabolic cart	Weight loss	REE was not a predictor of weight loss at 14 months (data not shown)	REE could not predict weight loss at 14 months	Low
Fidilio et al. (2021) [31] Spain	Total *N* = 39 (µ47 [SD: 12] y; 64% F; µ56.2 [SD: 5.6] kg/m^2^) Subgroup: OW NR Subgroup: OB NR	MBS assessment at 1-month and 12 months postoperative Rested, fasting state, supine position	Device: Vmax 29 (Sensor Medics, Yorba Linda, CA, USA), portable metabolic monitor	Weight loss	REE was not correlated with any weight-related variable including initial weight and weight at 1 and 12 months (data not shown)	REE could not predict weight loss up to 5-years post MBS	Low
Galtier et al. (2006) [32] France	Total *N* = 73 (µ 39 [SD: 10] y; 100% F; µ44.3 [SD: 7.0] kg/m^2^) Subgroup: OW *n* = 0 Subgroup: OB N/A	MBS preoperative assessment Rested, fasted state, supine position	Device: Deltatrac II (Datex Corp, Finland), ventilated hood IC	Weight loss	REE was not correlated with weight loss at 1 year (*r* = − 0.20, *p* = 0.09) RQ not correlated with weight loss at 1-year (r = −0.00, p > 0.05)	Neither REE nor RQ could predict weight loss at 1 year	Low
Hintze et al. (2021) [33] Canada	Total *N* = 36 (µ age NR [pre-menopausal]; 100% F; µ BMI NR [range 27 to > 40] kg/m^2^) Subgroup: OW NR Subgroup: OB NR	Pre- and post-weight loss (dietary) trial participants Rested, fasted state, supine position	Device: Vmax Encore 29 N (SensorMedics Corp, Yorba Linda, CA, USA), metabolic cart IC	Weight loss	Change in REE during intervention not correlated with weight loss at 10–12 weeks: *r* = − 0.092, *p* = NR	Change in REE could not predict weight loss	Low
Massarini et al. (2018) [7] Italy	Total *N* = 215 (µ61 [SD: 13] y; 68% F; µ33.3 [SD: 5.1] kg/m^2^) Subgroup: OW NR Subgroup: OB NR	Baseline assessment for weight loss (dietary–energy intake matched to REE) trial participants Rested, fasted state, supine position	Device: calorimeter metabolic cart (Sensor Medics, Italy)	Weight loss	Energy intake set on REE resulted in greater weight loss compared to estimated energy requirements (GEE estimate 2.143 [SE: 0.445] *p* < 0.001)	REE accurately measured energy requirement leading to greater weight loss over 1.5 years	Low
Wilms et al. (2018) [34] Switzerland	Total *N* = 233 (µ40 [SD: 11] y; 75% F; µ45.9 [SD: 6.4] kg/m^2^) Subgroup: OW NR Subgroup: OB NR	MBS preoperative assessment or stable reference group Rested, fasted state, supine position	Device: Deltatrac II (MBM 200, Hoyer, Bremen, Germany), ventilated hood IC	Weight loss	REE was associated with greater weight loss at 1 year (Q1: 1949 [SD: 401] kcal/day vs Q4: 2220 [SD: 446] kcal/d, *p* = 0.001) RQ was negatively associated with weight loss at 1 year (Q1: 0.84 [SD: 0.06] vs Q4: 0.82 [SD: 0.05], *p* = 0.016)	REE and RQ could predict weight loss at 1-year (higher REE = greater weight loss)	Low
Wang et al. (2008) [35] USA	Total *N* = 34 (µ59 [SD: 5] y; 100% F; BMI range 25–40 kg/m^2^) Subgroup: OW NR Subgroup: OB subgroup NR	Participants in a weight loss (dietary and exercise) trial Rested, fasted state	Device: MedGraphics CCM/D (MedGraphics Inc, Minneapolis, MN), metabolic cart with face mask	Post-intervention weight regain	Changes in REE over 20-week intervention were not correlated with future weight regain at 6 (*r* = 0.039, *p* = 0.831) or 12 months *r* = 0.044, *p* = 0.18)	Changes in REE during weight loss could not predict weight regain	Moderate

*CASP* Critical Appraisal Skills Programme, *ER* energy restriction, *F* female, *GEE* generalized estimating equations, *kcal/day* kilocalories per day, *M* male, *MBS* metabolic bariatric surgery, *NR* not reported, *SD* standard deviation, *OW* overweight, *OB* obese, *REE* resting energy expenditure, *RQ* respiratory quotient, *NR* not reported, *Q* quartile, *SD* standard deviation, *SE* standard error, *USA* United States of America, *VO*_*2*_ volume of oxygen consumption, *VCO*_*2*_ volume of carbon dioxide produced, *y* year, *µ* mean

#### Reliability of Indirect Calorimetry in Adults with Obesity

No studies reported on inter-rater reliability. Eight studies examined the intra-rater reliability (repeatability) of *n* = 10 devices: *n* = 7 standard desktop, *n* = 2 portable, and *n* = 1 whole-room IC devices (Table 2) with risk of bias ranging from low to moderate (Table 5). The standard desktop IC devices reported moderate-to-strong reliability in repeated REE measurements; however, studies either did not report statistical significance or were found to be not statistically significant [20, 22, 23, 37, 38]. The reliability of the COSMED Q-NRQ + applied via canopy versus mask could not be confidently interpreted due to the spurious effect of the ventilated or non-ventilated status of the two groups compared [40].

**Table 5:** Reliability of indirect calorimetry to determine basal metabolic rate or resting energy expenditure evaluated in adults with overweight or obesity (*n* = 7 studies)

Study	Sample	Assessment conditions	Indirect calorimetry	Intra-rater agreement^a^	Outcome	CASP risk of bias
Allerton et al. (2021) [36] USA	Total *N* = 35(µ36 [SD: 7] y; 32% F; 28–35 kg/m^2^) Subgroup: OW NR Subgroup: OB NR	Weight loss (medication) trial participants Fasted, rested state, supine position. Entered chamber for 3-days to control overnight and daily activities	Device: Four whole-room calorimeters (unspecified)	Repeated REE compared with ICC: ICC 93.2%, CV 3.7%, WSV 7388 kcal/d	Excellent reliability in repeated measures	Moderate
Anderson et al. (2014) [20] USA	Total *N* = 10 (µ52 [SD: 8] y; 74% F; µ31.8 [SD: 4.2] kg/m^2^)^b^ Subgroup: OW NR Subgroup: OB NR	Community sample Rested, fasted state, reclined position	Device: MedGem, handheld IC	Repeated REE compared with ICC: ICC 0.70 (95%CI 0.38–0.91), *p* = 0.15	Strong but inconsistent reliability in repeated measures (moderate retest reliability)	Low
			Device: Vmax 29 N (SensorMedics) IC, canopy hood, metabolic cart	Repeated REE compared with ICC: ICC 0.84 (95%CI 0.61–0.95), *p* = 0.65	Excellent reliability in repeated measures	Low
Bosy-Westphal et al. (2009) [37] Germany	Total *N* = 45 (µ33 [SD: 7] y; 100% F; µ35.9 [SD: 4.2] kg/m^2^) Subgroup: OW NR Subgroup: OB NR	Baseline and follow-up assessment for weight loss (dietary) trial participants Fasted state	Device: Vmax Spectra 29n (SensorMedics BV, Bilthoven, Netherlands, software Vmax version 12-1A), ventilated hood system	Repeated REE baseline: CV 5.2% Repeated REE follow-up after weight loss: CV 4.3%	Moderate reliability in repeated measures	Low
Frankenfield and Coleman (2013) [22] USA	Total *N* = 30 (µ44 [SD: 15] y; 84% F; µ33.4 [SD: 9.7] kg/m^2^)^a^ Subgroup: OW *n* = 0 Subgroup: OB *n* = NR (µ50 [SD: 12] y; %F NR; µ40.2 [SD: 6.2] kg/m^2^)^a^	Community sample Fasted, rested state, semi-recumbent position	Device: MedGem (Microlife, Golden, CO), handheld IC	REE in repeated measures: 1513 (SD: 303) vs 1509 (SD: 302) kcal/d; *p* = NR REE in repeated measures: 27% had second measure > 5% different from the first (limits of agreement − 166–162 kcal/d); 95%CI for the 73% of successful repeated measures as 55–86%, not reaching the 90% threshold of success to define precision REE in repeated measures via Bland–Altman: No bias (data NR)	Poor reliability between repeated measures	Low
	Total *N* = 100 (µ44 [SD: 15] y; 84% F; µ33.4 [SD: 9.7] kg/m^2^) Subgroup: OW *n* = 0 Subgroup: OB *n* = 60 (µ50 [SD: 12] y; %F NR; µ40.2 [SD: 6.2] kg/m^2^)	Community sample Fasted, rested state, supine or semi-recumbent position	Device: Deltatrac Monitor (Deltatrac MB101; Deltatrac, Yorba Linda, CA), canopy hood IC	REE in different positions compared via ANOVA: −50 (95%CI −73 to − 28), *p* = NR REE in different positions compared via Bland–Altman: no bias (data NR)	Reliable between postures (strength cannot be determined based on data provided)	Low
Nunes et al. (2022) [38] Portugal	Total *N* = 7 (µ43 [SD: 9] y; 34% F; µ31.1 [SD: 4.3] kg/m^2^)^a^ Subgroup: OW NR Subgroup: OB NR	Baseline assessment of weight loss (dietary) trial participants Fasted, rested state, supine position	Device: MedGraphics CPX Ultima (MedGraphics Corp, Breezeex Software, Italy), face-mask IC	REE in repeated measures had technical error measurement of 56.4 kcal, CV 4%, *p* = NR	Moderate repeatability in retests however no statistical significance provided	Low
Purcell et al. (2020) [27] Canada	Total *N* = 77 (median 30 [IQR: 26–37] y; 64% F; median 31.2 [IQR: 23.3–39.2] kg/m^2^) Subgroup: OW NR Subgroup: OB NR	Community sample and MBS sample (unclear if pre- or postoperative) Rested, fasted state, supine position	Device: Fitmate GS (COSMED, Chicago, IL, USA), canopy hood, portable IC device	REE in repeated measures compared via ICC: ICC 0.80 (95%CI 0.70–0.87), CV 8.6 (SD: 6.5) % Obesity subgroup analyses showed greater bias (14 kcal/day) than samples with healthy weight or overweight (− 28 kcal/d)	Strong repeatability in whole sample; but greater bias in sample with obesity	Low
Wu et al. (2019) [39] China	Total NR Subgroup: OW *n* = 94 (µ24 [SD: 3–3] y; 48% F; µ25.7 (M)–25.9 (F) [SD: 1.2–1.1] kg/m^2^) Subgroup: OB *n* = 64 (µ24-25 [SD: 2–3] y; 36% F; µ30.2 (M)–30.6 (F) [SD: 2.0–2.7] kg/m^2^)	Community sample Rested, fasted state, supine position	Device: MM2B gas analyzer (Cortex, Leipzig, Germany), portable metabolic IC system	REE in repeated measures in overweight were different: 1609 (SD: 224) kcal/d vs 1712 (SD: 224) kcal/d, *p* < 0.05 REE in repeated measures in obesity were different: 1778 (SD: 191) kcal/d vs 1885 (SD: 208) kcal/d, *p* < 0.05	Repeatability difficult to assess as measures of agreement not determined; and conditions for repeated measures were different (upon waking vs rest and positional differences)	Low
Winthrop et al. (2025) [40] USA	Total NA Subgroup: OW NR Subgroup: OB *n* = 4 (µ60 [SD: 6] y; %F NR; BMI > 30 kg/m^2^)	Post-cardiothoracic surgery with postoperative admission to the intensive care unit (critically ill) Minimal rested, fasted state (critically ill; fasting and no ambulation for ≥ 1 h), steady-state medically confirmed, position NR	Device: COSMED Q-NRQ + IC metabolic cart (COSMED, Rome, Italy) with canopy hood or mask	REE measured via the canopy (non-ventilated: 2054 kcal/d) was significantly higher than measured via the mask (ventilated: 1582 kcal/d), *p* = 0.010	Differences more likely a reflection of metabolic affect of ventilator dependency rather than use of mask versus canopy; cannot make confident interpretation on reliability of measurement techniques	Moderate

*CASP* Critical Appraisal Skills Programme, *CV* coefficient of variance/variation, *F* female, *kcal/d* kilocalories/day, *MBS* metabolic bariatric surgery, *NA* not applicable, *NR* not reported, *SD* standard deviation, *OW* overweight, *OB* obese, *REE* resting energy expenditure, *RQ* respiratory quotient, *USA* United States of America, *VO*_*2*_ volume of oxygen consumption, *VCO*_*2*_ volume of carbon dioxide produced, *WSV* within subject variance, *y* year, *µ* mean

^a^Intra-class correlation coefficient (ICC) often used for reliability testing. ICC of 1 represents perfect agreement, and a value of 0 means that there is no agreement at all. ICCs of 0 to 0.2 typically indicate poor agreement, 0.3 to 0.4 indicate fair agreement, 0.5 to 0.6 indicate moderate agreement, 0.7 to 0.8 indicate strong agreement, and > 0.8 indicates almost perfect agreement

^b^Repeated measures performed on subsample; characteristics presented for the full sample as not reported for the subsample

Although Wu et al. [39] reported statistically significant differences in repeated REE measurements for a worn-on-chest portable device, robustness of reliability was unable to be determined due to inconsistent conditions between the repeated measurements. The handheld MedGem device was found to have overall inconsistent and poor reliability [20, 22]. The whole-room IC demonstrated excellent reliability (ICC 93.2%, CV 3.7%); however, within subject variation was 7388 kcal/day [36].

## Discussion

This rapid review identified *n* = 22 articles that reported on the validity, predictive ability, and repeatability of REE measured by IC in adults with overweight and obesity. Although the risk of bias in individual studies was considered low to moderate with generally sound methodologies, confidence in the findings was weakened by several factors including imprecise measures and inadequate reporting of the results, particularly statistical significance. Standard desktop devices were the only type of IC evaluated across all outcomes, with inconsistent findings in concurrent validity (likely due to highly heterogenous comparator devices) and inconsistent predictive validity, but consistently good to strong reliability. Handheld IC devices were measured for concurrent validity and reliability, with weak findings for both outcomes. Whole-room IC was evaluated for reliability only, with excellent findings. However, a whole-room IC was also used as the reference standard for evaluating the concurrent validity of a standard IC device (Fitmate GS), where the two devices had poor agreement.

Standard desktop IC (i.e., metabolic carts) have been regarded for their validity and reliability in other patient samples and are often used as the reference standard when evaluating other REE assessment or prediction methods [15, 42–44]. The inconsistent findings for the concurrent validity of standard desktop ICs in this review may be due to poor choice of comparator devices, such as bioimpedance or accelerometers, which may be less accurate measures of REE as they do not use calibrated gas exchange. However, the single study that compared two standard-desktop IC devices found the KORR-MetaCheck and the Deltatrac-R3D had poor and imprecise findings. Despite being more practical and inexpensive compared to direct calorimetry or whole-room IC, standard desktop devices remain costly for most settings, are not easily portable, and require specialized training and patient preparation for proper administration [45–49]. The findings for predictive validity for weight outcomes were inconsistent, likely due to clinical heterogeneity in the types of outcomes assessed and the clinical group sampled. However, the finding that a standard desktop IC demonstrated that dietary energy intake prescriptions based on measured REE resulted in greater weight loss as compared to energy intake based on estimated equations is likely to have relevance for both weight loss interventional research and in the clinical setting and should be repeated to confirm results.

Although the more novel portable IC devices possess fewer feasibility limitations and have reported validity in samples without overweight or obesity [49–51], this review found consistently poor concurrent validity compared to standard desktop IC devices. Portable handheld ICs are susceptible to errors such as leaks in the mouthpiece, positional differences (i.e., muscle work to hold the mouthpiece), and use self-calibration rather than standard gas calibration [51, 52]. This was demonstrated in the included study by Frankenfield et al. [22], whereby different results were found when REE was measured in the supine versus semi-recumbent positions. Interestingly, the handheld MedGem device used in the semi-recumbent positioning had greater agreement with the Deltatrac used in the supine rather than semi-recumbent position. Another source of disagreement between handheld and standard desktop IC devices is the gases measured. Portable IC devices are required to use estimated rather than measured VCO_2_ in the modified Weir equation [20], which may lead to overestimation in higher extremes of BMI. Higher levels of adipose tissue produce more VO_2_, upon which the estimation of VCO_2_ is made [22, 53, 54]. Consistent with the findings of this review, which found the handheld MedGem may overestimate REE, the handheld MedGem device was found to report higher REE measures than the Harris–Benedict equation in adults with overweight or obesity [20].

This review found mixed results for the predictive ability of REE measured via IC for future weight change, with no consistent findings for device model nor follow-up timeframe. However, it should be noted that the only two studies reporting a significant finding were also those with the largest sample size [7, 34], suggesting those which reported insignificant results may have been underpowered. The inconsistent findings may also be due to the measurement of REE in only a fasted and rested state, which may not reflect meaningful daily fluctuations in REE influenced by varying lifestyle and medical factors [52]. It was interesting that dietary prescription based on REE measured via standard desktop IC was more useful for promoting weight loss than predictive equations. Despite limitations, this suggests the IC devices may have a clinical role regarding weight loss, especially if repeated measures can be made to account for changes in lifestyle and adaptive thermogenesis.

The whole-room IC demonstrated the strongest reliability with high ICC of 93.2%, aligning with other literature [55]. The controlled environment provided by a whole-room calorimeter minimizes external factors and may reduce discomfort and confinement stress, which is known to alter REE [56]. However, in adults with overweight and obesity, reliability has been measured only by a single study, decreasing confidence in the findings. While the reliability of standard desktop IC devices was not as strong as the whole-room IC, results were consistently moderate-to-strong in six studies, which increased confidence. The portable MedGem IC showed poor reliability in two studies. Handheld devices may be prone to handling errors and inter-operator variability, contributing to inconsistent measurements [23]. Frankenfield and Coleman et al. [22] found that repeat REE measurements were generally lower than initial measurements and aligned more closely with standard IC-derived REE. This may reflect a bias due to researchers becoming more proficient with device handling and increased participant familiarization. The poor reliability findings for portable IC devices suggest caution should be used in clinical settings as it may be unsuitable for precise tracking of metabolic changes over time.

In situations where precise measurement of REE could confer morbidity and mortality benefits such as critically ill patients in ICU, there could be a role for standard/desktop-based IC due to superior reliability and potential validity. Conversely, handheld IC devices may provide a valuable alternative for community-based measurement of REE in situations where portability, ease of use, and feasibility are paramount and precision less critical. In a research setting where precision and reliability are of primary concern with no feasibility constraints, whole-room IC may be the most appropriate. It is important to note that making specific recommendations regarding widespread adoption and application of IC across a range of clinical settings was beyond the scope of this paper due to the limitations in the body of research identified. Additionally, future research of higher quality may lead to increased confidence in the application of IC devices in the clinical setting.

### Strength and Limitations

The strengths of the rapid review include strong methodological rigor, with key review steps completed in duplicate, however, was limited by the risk of missing eligible studies by searching only two literature databases. The results reported in this review were limited by poor reporting in the original studies, including overly brief descriptions of measurements, failing to fully report results, failing to conduct and/or report the statistical significance of findings, and failing to consistently adhere to consistent protocols to establish and confirm a resting environment and steady state. Conclusions of this review are not only limited by small study numbers for some outcomes, but by extreme clinical heterogeneity in comparator devices and reference standards, including the use of non-IC devices, rendering it difficult to compare the studies. Therefore, guidance for researchers is needed to select robust and feasible reference standards to evaluate the concurrent validity of IC devices given the limitations of current methods and extreme heterogeneity used in the body of evidence, which limits the synthesis of findings and clinical interpretation.

## Conclusion

Indirect calorimetry measures of REE have been insufficiently evaluated for validity and reliability in adults with overweight and obesity, with the existing studies often poorly reported and lacking in appropriate psychometric design. Standard desktop IC devices have inconsistent concurrent and predictive validity but moderate-to-strong reliability. Handheld portable IC devices lack concurrent validity and reliability and were not assessed for predictive validity. Whole-room IC devices had excellent reliability but were not assessed for validity.

## Supplementary Information

Below is the link to the electronic supplementary material.

Supplementary file 1 (DOCX 78.9 KB)

## Data Availability

No datasets were generated or analysed during the current study.
