# Supplementary material for: Validity and Reliability of Resting Energy Expenditure Measured by Indirect Calorimetry in Adults with Overweight and Obesity: a Rapid Systematic Review
Source: Obes Surg. 2025 Sep 9;35(10):4492–507. doi: 10.1007/s11695-025-08220-w (PMC12540577; doi:10.1007/s11695-025-08220-w)
Supplement: Supplementary file 1 — (DOCX 78.9 KB) [file 11695_2025_8220_MOESM1_ESM.docx]

**Table S1**: Table of terms used in search strategy for PubMed and Web of Science.

| **Database searched** | **Search strategy** |
| --- | --- |
| PubMed | ((((((calorimetry, respiration[MeSH Terms]) OR (indirect calorimetry[MeSH Terms])) OR (nutrition assessment[MeSH Terms])) OR (basal metabolism[MeSH Terms])) OR (energy metabolism[MeSH Terms])) OR ((((((((((indirect calorimetry[Title/Abstract]) OR (nutrition assessment[Title/Abstract])) OR (basal metabolism[Title/Abstract])) OR (energy metabolism[Title/Abstract])) OR (resting energy expenditure[Title/Abstract])) OR (prognostic nutrition index[Title/Abstract])) OR (ree[Title/Abstract])) OR (caloric intake[Title/Abstract])) OR (basal metabolic rate[Title/Abstract])) OR (BMR[Title/Abstract]))) AND (((((reproducibility of results[MeSH Terms]) OR (diagnostic errors[MeSH Terms])) OR (sensitivity and specificity[MeSH Terms])) OR (predictive value of tests[MeSH Terms])) OR (((((((((((((reproducibility of results[Title/Abstract]) OR (diagnostic errors[Title/Abstract])) OR (specificity[Title/Abstract] AND sensitivity[Title/Abstract])) OR (predictive value of tests[Title/Abstract])) OR (reproduc*[Title/Abstract])) OR (reliab*[Title/Abstract])) OR (variability[Title/Abstract])) OR (diagnostic[Title/Abstract])) OR (accuracy[Title/Abstract])) OR (validity[Title/Abstract])) OR (agreement[Title/Abstract])) OR (probability[Title/Abstract])) OR (youden's index[Title/Abstract])))) AND (((abdominal obesity[MeSH Terms]) OR (overweight[MeSH Terms])) OR ((((((((abdominal obesity[Title/Abstract]) OR (obese[Title/Abstract])) OR (obesity[Title/Abstract])) OR (overweight)) OR (metabolic syndrome[Title/Abstract])) OR (excess weight[Title/Abstract])) OR (BMI[Title/Abstract])) OR (adiposity[Title/Abstract]))) |
| Web of Science | “indirect calorimetry” OR “nutrition assessment” OR “basal metabolism” OR “energy metabolism” OR “resting energy expenditure” OR “prognostic nutrition index” OR “REE” OR “caloric intake” OR “basal metabolic rate” OR “BMR” AND “reproducibility of results” OR “diagnostic errors” OR “sensitivity and specificity” OR “predictive value of tests” OR “reproducibility of results” OR “diagnostic errors” OR reproduce* OR reliab* OR variability OR diagnostic OR accuracy OR validity OR agreement OR probability OR “Youden’s index” AND “abdominal obesity” OR “overweight” OR “obese” OR “obesity” OR “overweight” OR “metabolic syndrome” OR “excess weight” OR “BMI” OR “adiposity” |

**Identification of studies via databases and registers**

Records removed *before screening*:

Duplicate records removed in EndNote via automation (n = 204)

Duplicates identified by automation software in Covidence (n = 62)

Duplicates identified manually (n=5)

Records identified from:

Web of Science (n = 2,109)

PubMed (n = 3118)

**Total n = 5,227**

**Identification**

Records screened

(n = 4,956)

Records excluded

(n = 4,376)

**Screening**

Reports sought for retrieval

(n = 580)

Reports not retrieved

(n = 0)

Reports assessed for eligibility

(n = 580)

Reports excluded:

Wong population (n = 309)

Wong comparator (n = 149)

Wrong outcomes (n = 52)

Wrong RMR measure (n = 38)

Non-English (n = 17)

Wrong study design (n = 12)

Conference abstract (n = 2)

Wrong intervention (n = 1)

Studies included in review

(n = 22)

**Included**

**Figure 1**: PRISMA flow diagram of the literature search and study selection

**Table S2:** Assessment of study quality and risk of bias according to the Critical Appraisal Skills Programme (CASP) Diagnostic Checklist

| **Study** | **Was there a clear research question for the study to address?** | **Was there a comparison with an appropriate reference standard?** | **Did all the patients get the diagnostic test and the reference standard?** | **Could the results of the test have been influenced by the results of the reference standard?** | **Is the disease status of the tested population clearly described?** | **Were the methods for performing the test described in sufficient detail?** | **What are the results (are the results presented)?** | **How sure are we about the results? Consequences and costs of alternatives performed?** | **Can the results be applied to your patients / population of interest?** | **Can the test be applied to your population or patient of interest?** | **Were all outcomes important to the individual or population considered?** | **What would be the impact of using this test on your patients or population?** |
| --- | --- | --- | --- | --- | --- | --- | --- | --- | --- | --- | --- | --- |
| **Cooper 2009** | Yes | Yes | Yes | No | Yes | Yes^a^ | Yes | N/A | Can't tell | Yes | Yes | N/A |
| **Popp 2020** | Yes | Yes | Yes | No | Yes | Yes | Yes | NA | Yes | Yes | Yes | N/A |
| **Galtier 2006** | Yes | No | No | No | Yes | Yes | Yes | N/A | Yes | Yes | Yes | N/A |
| **Massarini 2018** | Yes | Yes | No | No | Yes | Yes | Yes | N/A | Yes | Yes | Yes | N/A |
| **Allerton 2021** | Yes | No | No | No | No | Yes | Yes | N/A | Yes | Can't tell | Yes | N/A |
| **Bentes 2021** | Yes | Yes | Yes | No | Yes | Yes | Yes | N/A | No | Yes | Yes | N/A |
| **Wilms 2018** | Yes | Yes | Yes | No | Yes | Yes | Yes | N/A | Yes | Yes | Yes | N/A |
| **Elbelt 2010** | Yes | Yes | No | No | Yes | Yes | Yes | N/A | Yes | Yes | Yes | N/A |
| **Wang 2008** | Yes | No | No | No | Yes | Yes | Yes | N/a | No | Yes | Yes | N/A |
| **De Oliveira 2012** | Yes | Yes | Can't tell | No | Yes | Yes | Yes | N/A | Yes | Yes | Can't tell | N/A |
| **Hintze 2021** | Yes | No | No | No | Yes | Yes | Yes | N/A | Yes | Yes | Yes | N/A |
| **Wu 2019** | Yes | No | No | No | Yes | Yes | Yes | N/A | Yes | Yes | Yes | N/A |
| **Purcell A 2020** | Yes | Yes | Yes | No | Yes | Yes | Yes | N/A | Yes | Yes | Yes | N/A |
| **Bosy-Westphal 2009** | Yes | Yes | Yes | No | Yes | Yes | Yes | N/a | Yes | Yes | Yes | N/A |
| **Filidio 2021** | Yes | Yes | Yes | No | Yes | Yes | Yes | N/A | Yes | Yes | Yes | N/A |
| **Frankenfield 2013** | Yes | Yes | Yes | No | No | Yes | Yes | N/a | Can't tell | Yes | Yes | N/A |
| **Baudrand 2013** | Yes | Yes | Can't tell | No | Yes | No | Yes | N/A | Yes | Yes | Yes | N/A |
| **Anderson 2014** | Yes | Yes | Yes | No | Yes | Yes | Yes | N/A | Yes | Yes | Yes | N/A |
| **Purcell B 2020** | Yes | Yes | Yes | No | Yes | Yes | Yes | N/A | Yes | Yes | Yes | N/A |
| **Das 2003** | Yes | No | No | No | Yes | Yes | Yes | N/A | Yes | Yes | Yes | N/A |
| **Nunes 2022** | Yes | Yes | Can't tell | No | Yes | Yes | Yes | N/A | Can't tell | Yes | Yes | N/A |
| **Winthrop** | Yes | No | No | No | Yes | Yes | Yes | N/A | Yes | Yes | Yes | N/A |

**a.** Reported in a subsequent publication.

**Table S3:** Descriptions of indirect calorimeter devices and comparator devices used to evaluate the concurrent validity of indirect calorimetry to determine basal metabolic rate or resting energy expenditure evaluated in adults with overweight or obesity

| **Study** | **Indirect calorimetry devices** | **Comparator devices** |
| --- | --- | --- |
| **Concurrent validity of a portable hand-held indirect calorimeter** | | |
| Anderson et al 2014[19]  USA | **Device:** MedGem  **Description:** Handheld IC, two sensors which measure O_2_ and air flow.  **Calibration:** NR.  **Measure:** Measures VO_2_ for determination of REE and an RQ constant of 0.85. | **Device:** Vmax 29N (SensorMedics) IC.  **Description:** Canopy hood, metabolic cart.  **Calibration:** Calibrated before use according to manufacturer instructions.  **Measure:** O_2_ consumption and CO_2_ production measured for ≥20-minutes. REE and RQ calculated from substrate oxidation rates. |
| Cooper et al 2009[20]  USA | **Device:** MedGem (Microlife USA, Golden, CO).  **Description:** Handheld device utilizing nose-clip, measures VO_2_ on a dual-channel oxygen sensor; does not measure VCO_2_.  **Calibration:** Auto-calibrated.  **Measure**: Nose-clip. Up to 10-minute assessment, first 2-minutes discarded. Stead-state required; if not achieved the average of minutes two and 10 are utilized. REE calculated assuming an RER of 0.85 with modified Weir equation. | **Device:** Deltatrac II Metabolic Monitor (VIASYS Healthcare, Inc., SensorMedics, Yorba Linda, CA)  **Description:** Device no longer available; was previously used as standard measure in practice.  **Calibration:** Calibrated to reference gases.  **Measure**: 20-minute assessment, first 5-minutes were discarded. REE calculated. |
| Frankenfield & Coleman, 2013[21]  USA | **Device:** MedGem (Microlife, Golden, CO).  **Description:** Handheld indirect calorimeter, mouthpiece with nose clip.  **Calibration:** Self-calibration before each measurement.  **Measure:** VO_2_ measured; automatically detects steady state to time measurements. REE determined with Weir equation, using RQ of 0.85. Semi-recumbent position only. | **Device:** Deltatrac Monitor (Deltatrac MB101; Deltatrac, Yorba Linda, CA).  **Description:** Open circuit indirect calorimeter, clear plastic canopy, metabolic cart.  **Calibration:** Calibration of gas sensors conducted before each session with standardized gas.  **Measure:** 15-minute assessment, first 5-minutes discarded. VO_2_ and VCO_2_ measured. CV had to remain <10%. REE determined with Weir equation. Supine and semi-recumbent position measures. |
| Purcell et al 2020 [22]  Canada | **Device:** MedGem (Microlife Medical Home Solutions, Inc, San Jose, CA, USA)  **Description:** Portable indirect calorimeter, mouthpiece with nose-clip.  **Calibration:** Self-calibrated.  **Measure:** REE based on O_2_ measured for 5-10-minutes until steady state achieved; CO_2_ assumed based on an RER of 0.85. | **Device:** Vmax 29 N (Senor-Medics, Yorba Linda, CA, USA)  **Description:** Metabolic cart, ventilated hood.  **Calibration:** Manual calibration of the flow meter before testing with a 3 L syringe. Gas analyzers were automatically calibrated prior to each test using standardized gas.  **Measure:** REE determined using the abbreviated Weir equation using measured O_2_ and CO_2_. Included only minutes in which variations in gas volumes varied ≤10% over the preceding 5-minutes. |
| **Concurrent validity of a standard indirect calorimeter** | | |
| De Oliveira et al 2012[23]  Brazil | **Device:** KORR-MetaCheck device (Metabolic Rate Analysis System, model 7100, KORR Medical Technologies).  **Description:** Portable indirect calorimeter.  **Calibration:** NR.  **Measure:** 10-15 minutes. REE calculated using Weir formula. | **Device:** Deltatrac-R3D (Deltatrac II, MBM-200, Datex Instrumentarium Corporation)  **Description:** Metabolic cart.  **Calibration:** NR.  **Measure:** 30-minute assessment. REE calculated using Weir formula. |
| Bentes et al 2021[24]  Brazil | **Device:** Fitmate Pro (COSMED, Rome, Italy)  **Description:** Portable device for use during rest or exercise with mask.  **Calibration:** NR.  **Measure:** Turbine flowmeter applied for 15-minutes in steady state, measuring galvanic fuel cell oxygen sensor to analyze expired gases. Standard metabolic formulas calculate oxygen uptake with EE calculated using a fixed RQ of 0.85. | **Device:** InBody 720 (Biospace, Seoul, Korea).  **Description:** Octopolar bioimpedance.  **Calibration:** NR  **Measure:** Measures body mass, fat mass, skeletal muscle mass, fat percentage, fat-free mass, visceral fat area, and RREEMR. |
| Popp et al 2020[25]  USA | **Device:** COSMED Quark RMR metabolic cart (COSMED, Rome, Italy).  **Description:** Open circuit-IC with flow-dilution canopy hood.  **Calibration:** Flow meter and gas-analyzer calibrated as per manufacturer instructions.  **Measure:** VO_2_ consumption and VCO_2_ production measured every 10-seconds for 20-minutes following a 5-minute run-in stabilization period. REE calculated with Weir equation at steady-state in via averages of 5-minute intervals. RQ calculated as VCO_2_/ VO_2_. | Range of RQ compared to the physiological range. |
| Purcell et al 2020[26]  Canada | **Device:** Fitmate GS (COSMED, Chicago, IL, USA)  **Description:** Canopy hood, portable device.  **Calibration:** Self-calibrated before each test; flow meter manually calibrated once per week as per manufacturer instructions.  **Measure:** REE calculated using Weir equation based on measured O_2_ and assumed CO_2_ using an RQ of 0.85. | **Device:** Whole body indirect calorimeter.  **Description:** Open circuit, controlled atmosphere.  **Calibration:** Full calibration once a week; abbreviated calibration prior to each test.  **Measure:** REE calculated via Weir equation based on measured O_2_ and CO_2_ for 1-hour, first 30-minutes discarded. |
| Baudrand et al 2013[27]  Chile | **Device:** Deltatrac MBM-100 (Datex Instrumentarium Corp, Helsinki, Finland).  **Description:** Indirect calorimeter.  **Calibration:** NR.  **Measure:** REE. | **Device:** OMRON HBF 500 (Omron Corp, Kyoto, Japan)  **Description:** Body composition monitor.  **Calibration:** NR.  **Measure:** REE. |
| Elbelt et al 2010[28]  Germany | **Device:** Deltatrac II (Datex-Ohmeda, Freiburg, Germany)  **Description:** Ventilated hood.  **Calibration:** Gas analyzers were calibrated to standardized gas before each measurement adapted to body weight.  **Measure:** REE calculated according to Consolazio. | **Device:** SenseWear armband accelerometer (InnerView Professional, Version 6.1, SMT medical technology, Wuerzburg, Germany).  **Description:** Multisensor array including 2-axis accelerometer.  **Calibration:** NR.  **Measure:** Worn on upper right arm for 5-10-minutes before measurement of REE. |

**Table S4:** Descriptions of indirect calorimeter devices used to evaluate the predictive ability of indirect calorimetry to determine basal metabolic rate or resting energy expenditure evaluated in adults with overweight or obesity

| **Study** | **Indirect calorimetry devices** |
| --- | --- |
| Das et al 2003[29]  USA | **Device:** Deltatrac (SensorMedics Corp, Yorba Linda, CA)  **Description:** Portable metabolic cart.  **Calibration:** Calibrated with standard gas mixture before each test. Standard alcohol burn tests were conducted periodically.  **Measure:** VO_2_ and VCO_2_ were measured every 1-minute for 30-minutes. REE calculated according to Weir’s equation with mean of VO_2_ and VCO_2_ values. |
| Fidilio et al 2021[30]  Spain | **Device:** Vmax 29 (Sensor Medics, Yorba Linda, CA, USA)  **Description:** Portable metabolic monitor.  **Calibration:** Calibrated prior to each measurement.  **Measure:** 15-20-minute measurement, first 5-minutes discarded. VO_2_ consumption, VCO_2_ production, RQ, and REE measured. |
| Galtier et al 2006[31]  France | **Device:** Deltatrac II (Datex Corp, Finland)  **Description:** Ventilated hood.  **Calibration:** Adjusted for temperature and barometric pressure, calibrated with standardized gas mixture every morning.  **Measure:** RQ and REE calculated according to Ferrannini. |
| Hintze et al 2021[32]  Canada | **Device:** Vmax Encore 29N (SensorMedics Corp, Yorba Linda, CA, USA).  **Description:** Metabolic cart.  **Calibration:** Ethanol burning test, calibrated against standardized gas mixture daily  **Measure:** CO2 and O2 measured for 30 minutes, last 20 minutes were used in REE calculation. Change in REE assessed pre- and post-intervention. |
| Massarini et al 2018[7]  Italy | **Device:** Calorimeter metabolic cart (Sensor Medics, Italy)  **Description:** Open circuit, canopy hood, flow rate directly measured with a digital turbine flowmeter.  **Calibration:** Certified 3-L calibration syringe, span, and delay alignment of gas analyzers before each test using certified calibration gas.  **Measure:** REE using the last 15-20 minutes of measurements corresponding to steady state, minute-by-minute reading of VO_2_ and VCO_2_. RQ ratio of VCO_2_ and VO_2_. |
| Wilms et al 2018[33]  Switzerland | **Device:** Deltatrac II (MBM 200, Hoyer, Bremen, Germany)  **Description:** Ventilated hood system.  **Calibration:** Gas analyzer calibrated before each measure with standardized gas.  **Measure:** 20-minute assessment. O_2_ and CO_2_ measured every minute and converted to REE using abbreviated Weir’s equation. RQ estimated as ratio of CO_2_ production to O_2_ uptake. |
| Wang et al 2008[34]  USA | **Device:** MedGraphics CCM/D (MedGraphics Inc, Minneapolis, MN).  **Description:** Metabolic cart, face mask, one way valve  **Calibration:** NR.  **Measure:** 30-minute assessment, steady state defined as ±5% in oxygen consumed and rate of exchange ratio. REE measured in steady state at baseline and end of intervention (20-weeks). |

**Table S5:** Descriptions of indirect calorimeter devices used to evaluate reliability of indirect calorimetry to determine basal metabolic rate or resting energy expenditure evaluated in adults with overweight or obesity (n=7 studies)

| **Study** | **Indirect calorimetry device** |
| --- | --- |
| Allerton et al 2021[35]  USA | **Device:** Four whole-room calorimeters (unspecified)  **Description:** Room volume of 27m^3^ or 30m^3^ with environmental control.  **Calibration:** Standardized across sites.  **Measure:** O_2_ and CO_2_ concentration measured every 10-seconds. REE calculated by the measurement of VO_2_ and VCO_2_. Measures repeated for 3-consecutive days. |
| Anderson et al 2014[19]  USA | **Device:** MedGem  **Description:** Handheld IC, two sensors which measure O_2_ and air flow.  **Calibration:** NR.  **Measure:** Measures VO_2_ for determination of REE and an RQ constant of 0.85. Repeated within 5-days. |
|  | **Device:** Vmax 29N (SensorMedics) IC.  **Description:** Canopy hood, metabolic cart.  **Calibration:** Calibrated before use according to manufacturer instructions.  **Measure:** O_2_ consumption and CO_2_ production measured for ≥20-minutes. REE and RQ calculated from substrate oxidation rates. |
| Bosy-Westphal et al 2009[36]  Germany | **Device:** Vmax Spectra 29n (SensorMedics BV, Bilthoven, Netherlands, software Vmax version 12-1A)  **Description:** Ventilated hood system.  **Calibration:** Flow calibration performed with 3-L syringe and gas analyzers were calibrated before and every 5-minutes during assessment.  **Measure:** Data collected every 20-seconds for ≥45-minutes (first 10-minutes discarded). O_2_ and CO_2_ volume converted to REE using an abbreviated Weir equation. Measure repeated on different mornings at baseline and at follow-up. |
| Frankenfield & Coleman, 2013[21]  USA | **Device:** MedGem (Microlife, Golden, CO).  **Description:** Handheld indirect calorimeter, mouthpiece with nose clip.  **Calibration:** Self-calibration before each measurement.  **Measure:** VO_2_ measured; automatically detects steady state to time measurements. REE determined with Weir equation, using RQ of 0.85. Semi-recumbent position only; repeated measures in same session within 1 minute of terminating first measurement with no change in subject posture |
|  | **Device:** Deltatrac Monitor (Deltatrac MB101; Deltatrac, Yorba Linda, CA).  **Description:** Open circuit indirect calorimeter, clear plastic canopy.  **Calibration:** Calibration of gas sensors conducted before each session with standardized gas.  **Measure:** 15-minute assessment, first 5-minutes discarded. VO_2_ and VCO_2_ measured. CV had to remain <10%. REE determined with Weir equation. Supine versus semi-recumbent position measures. |
| Nunes et al 2022[37]  Portugal | **Device:** MedGraphics CPX Ultima (MedGraphics Corp, Breezeex Software, Italy).  **Description:** Face-mask measure.  **Calibration:** NR.  **Measure:** Breath-by-breath analysis of VO_2_ and VCO_2_ in steady-state for 30 minutes; excluding first and last 5-minutes of measurement. REE calculated using the Weir equation. Lowest REE utilized for analysis. Repeated measures in same session. |
| Purcell et al 2020[26]  Canada | **Device:** Fitmate GS (COSMED, Chicago, IL, USA)  **Description:** Canopy hood, portable device.  **Calibration:** Self-calibrated before each test; flow meter manually calibrated once per week as per manufacturer instructions. 2-7 days between repeated measures.  **Measure:** REE calculated using Weir equation based on measured O_2_ and assumed CO_2_ using an RQ of 0.85. |
| Wu et al 2019[38]  China | **Device:** MM2B gas analyzer (Cortex, Leipzig, Germany)  **Description:** Portable metabolic system, breath-by-breath, worn on chest with harness.  **Calibration:** Calibrated prior to each test according to manufacturer instructions.  **Measure:** REE determined by measuring averages of VO_2_ and VCO_2_ for 15-minutes, first 5-minutes discarded. Measured upon waking whilst supine and repeated half an hour later whilst sitting up. |
| Winthrop et al 2025 [39]  USA | **Device**: COSMED Q-NRQ+ metabolic cart (COSMED, Rome, Italy)  **Description**: Metabolic cart with canopy or mask.  **Calibration**: NR.  **Measure**: REE calculation NR. First measure taken within 72-hours post-surgery; subsequent measures every 5-7-days during ICU admission. REE measures averaged for each patient. Measured in ventilated state (via mask) or non-ventilated state (canopy). |
